# A comparative study reveals the relative importance of prokaryotic and eukaryotic proton pump rhodopsins in a subtropical marginal sea

**DOI:** 10.1038/s43705-023-00292-y

**Published:** 2023-08-18

**Authors:** Minglei Ma, Hongfei Li, Cong Wang, Tangcheng Li, Jierui Wang, Huatao Yuan, Liying Yu, Jingtian Wang, Ling Li, Senjie Lin

**Affiliations:** 1 State Key Laboratory of Marine Environmental Science, College of Ocean and Earth Science, Xiamen University, Xiamen, 361102 China; 2 National Engineering Research Center for Marine Aquaculture, Zhejiang Ocean University, Zhoushan, 316022 China; 3 Biology Department and Institute of Marine Sciences, College of Science, Shantou University, Shantou, 515063 China; 4 Central Laboratory, the Second Affiliated Hospital of Fujian Medical University, Quanzhou, 362000 China; 5 Laboratory of Marine Biology and Biotechnology, Qingdao National Laboratory of Marine Science and Technology, Qingdao, 266237 China; 6 Department of Marine Sciences, University of Connecticut, Groton, CT 06340 USA

**Keywords:** Microbial ecology, Environmental microbiology

## Abstract

Proton-pump rhodopsin (PPR) in marine microbes can convert solar energy to bioavailable chemical energy. Whereas bacterial PPR has been extensively studied, counterparts in microeukaryotes are less explored, and the relative importance of the two groups is poorly understood. Here, we sequenced whole-assemblage metatranscriptomes and investigated the diversity and expression dynamics of PPR in microbial eukaryotes and prokaryotes at a continental shelf and a slope site in the northern South China Sea. Data showed the whole PPRs transcript pool was dominated by Proteorhodopsins and Xanthorhodopsins, followed by Bacteriorhodopsin-like proteins, dominantly contributed by prokaryotes both in the number and expression levels of PPR unigenes, although at the continental slope station, microeukaryotes and prokaryotes contributed similarly in transcript abundance. Furthermore, eukaryotic PPRs are mainly contributed by dinoflagellates and showed significant correlation with nutrient concentrations. Green light-absorbing PPRs were mainly distributed in >3 μm organisms (including microeukaryotes and their associated bacteria), especially at surface layer at the shelf station, whereas blue light-absorbing PPRs dominated the <3 μm (mainly bacterial) communities at both study sites, especially at deeper layers at the slope station. Our study portrays a comparative PPR genotype and expression landscape for prokaryotes and eukaryotes in a subtropical marginal sea, suggesting PPR’s role in niche differentiation and adaptation among marine microbes.

## Introduction

Rhodopsins are now known in all three domains of life. The best known is sensory rhodopsin for vision in animal eyes. More functionally diverse rhodopsins occur in microbial organisms (microbial rhodopsins) [1]. Initial discoveries of microbial rhodopsins date back to the 1970s, when rhodopsins in *Halobacterium halobium* were characterized as proton or chloride pumps [2–4]. After a two-decade quiescent period, interest in microbial rhodopsins was rekindled by the discovery of proton-pump rhodopsins (PPRs), a subfamily of microbial rhodopsins, in the SAR86 clade [5] and many other bacteria in the surface ocean. PPRs pump protons from the cytoplasm out extracellularly and create a proton gradient that has the force to drive ATP production [6]. These photoenergy capturing rhodopsins were widely reported to occur in 48% of small-size particles (<0.8 μm) in the ocean’s photic zone [7, 8] or in 13–70% of bacteria living in the surface ocean [9, 10]. They are now known to be abundantly distributed globally, from the aquatic system (including marine and fresh-water systems) to edaphic systems [11], from the tropic [12] to polar regions [13, 14], and taxonomically, from giant virus and eubacterial organisms [2, 14–16] to eukaryotic microbes [17, 18].

Most of the proton-pump microbial rhodopsins documented so far are outward proton pumps, which function to produce ATP in the cells, although inward proton pump rhodopsins (i.e. xenorhodopsins and schizorhodopsins) have also been reported [19–21]. For that reason and brevity, the term PPRs will be used from here on to depict microbial rhodopsins that were found to be outward proton pump rhodopsins, the focus of the present study. PPRs found so far include proteorhodopsins (PRs) [22–24], bacteriorhodopsins (BRs) [25], xanthorhodopsins (XRs) [26, 27], *exiguobacterium* rhodopsins (ESRs) [28] and actinorhodopsins (ActRs) [29]. As mentioned above, PPRs can hyperpolarize the membrane potential, which could synthesize ATP to benefit the PPR-containing microorganisms [30]. However, the ecological role of the diverse PPRs is not completely clear, even though studies have generally shown that they promote the growth or survival of their carrier microbes in nutrient-poor environments [24, 31]. In dinoflagellates, PPRs may provide energy to support growth under food or nutrient-limited or light-limited conditions [32, 33]. In diatoms, PPRs have been implicated in coping with iron limitation [18].

The relative importance of prokaryotic PPRs and eukaryotic PPRs in the ocean is poorly understood, both in terms of each group’s contribution to the total diversity and the total expression (potential activity) of PPRs. Only a limited number of studies have documented expressed PPRs from microeukaryotes in the natural marine environment, which mainly focused on dinoflagellates [17], diatoms [18], and other microbial eukaryotes [13]. Thanks to the increasing accessibility of high throughput sequencing, metatranscriptomic studies on dinoflagellate blooms have revealed high diversities and high expression of PPRs in the bloom species *Prorocentrum shikokuense* (formerly *Prorocentrum donghaiense*), suggesting that PPRs might function to fuel the blooms in dim light or phosphorus nutrient-limited environments [34, 35]. The differential distribution of PPR between eukaryotes and prokaryotes and the relative expression levels of prokaryotic and eukaryotic PPRs remains underexplored, however. A recent study measured retinal concentration as the proxy of PPR abundance in a Mediterranean-Atlantic Ocean transect and found that the microbial PPRs there were primarily contributed by bacteria; they estimated that prokaryotic PPRs could absorb as much light energy as chlorophyll-*a*-based phototrophy, sufficient to sustain bacterial basal metabolism in the oligotrophic seas [36].

Furthermore, point mutations are known to alter absorption maxima in PPRs. Therefore, some PPRs (i.e. proteorhodopsins, PRs) absorb green-light (GPRs, λmax = 525 nm) and others absorb blue-light (BPRs, λmax = 490 nm) [37, 38]. Spectral tuning of PPRs from blue to green light is due to the substitution of one amino acid residue in the retinal pocket. At position 105 of the typical PPR, it is a methionine or leucine in GPR but is the polar glutamine in BPR [24, 39, 40]. A new study found that in the two PRs named ISR34 and ISR36, besides position 105, Cys189 is a vital residue controlling spectral tuning [41]. How these two types of PPRs are distributed spatially and taxonomically in the ocean is not well understood.

This study addresses the above-mentioned research gaps by using metatranscriptome sequencing. The data were used to analyze the diversity and relative expression levels of PPRs, in both prokaryotes and eukaryotes, in the northern South China Sea (NSCS), a subtropical marginal sea.

## Materials and methods

### Sample collection and environmental measurements

Seawater samples were collected at two sites, the continental shelf station (C6, 117.46°E, 22.13°N) and the continental slope station (C9, 117.99°E, 21.69°N) (Fig. S1), of Taiwan Strait onboard the research vessel Yanping II from 6th to 12th of August 2016. Samples were collected at the surface (SUR), deep chlorophyll maximum (DCM) layer, and bottom of the photic zone (BOT) using Seabird CTD (conductivity-temperature-depth profiler) rosette equipped with 12-liter Niskin bottles. For each sample, 20–60 liters (details in Supplementary 2) of seawater were pre-filtered through 200 μm to remove large organisms and then serially filtered onto a 3 μm and a 0.22 μm pore-size, 142 mm diameter, polycarbonate membrane (Merck Millipore, MA, USA). The filters were immediately transferred to 2 mL tube (KIRGEN, Shanghai, China) immersed with TRIzol regent buffer and stored in liquid nitrogen during the cruise and then stored at −80 °C in the lab until RNA extraction. Two replicate samples were collected from each sampling event. Totally, 20 samples for transcriptome analysis were collected. Twenty samples for 16 S and 18 S rRNA gene (rDNA SSU) analysis were collected in same way as RNA samples but were immersed in DNA lysis buffer before being stored at −80 °C. The analysis of the DNA samples to characterize the microbial community structures has been reported elsewhere [42, 43].

Bacteria in the small (0.22–3 μm) fraction are considered free-living, whereas those in the large (3–200 μm) fraction are considered particle-associated. Although we could not rule out the possibility that some free-living bacteria might be retained in the large fraction when the membrane was clogged by accumulated biomass, we think it is unlikely that this “accidental” bacteria would dominate over the authentic particle-associated bacteria, particularly because the biomass in the oceanic stations was generally low. Temperature, salinity, and turbidity were measured using CTD (SBE 17plus V2, Sea-Bird Scientific, Bellevue, WA, USA) at each sampling event. The concentrations of dissolved inorganic nitrogen (DIN) (nitrate and nitrite), silicate and phosphate were measured using a Technicon AA3 Auto-Analyzer (Bran-Lube, Norderstedt, Germany), and all measurements were performed on triplicate samples.

### RNA extraction and Illumina high-throughput sequencing

RNA was extracted from the field samples following our previously reported protocol [34] that combines the TRIzol procedure with Zymo RNA purification column, and incorporates FastPrep-24 bead mill (MP Biomedicals, Solon, USA) with 0.5 mm- and 0.1 mm-diameter zirconia/silica beads (Biospec, Shanghai, China) to completely break the cells. The RNA purity and quantity were assessed using NanoDrop 2000 (Thermo Scientific, Waltham, MA, USA) and Agilent 2100 Bioanalyzer (Agilent Technologies, Palo Alto, CA, USA). The RNA samples with RNA integrity number (RIN) ≥ 6.0 were used for RNA-seq sequencing (BGI, Shanghai, China). Ribosomal RNA of each sample (1 μg RNA) was removed using a Ribo-Zero™ rRNA Removal Kit (Human/Mouse/Rat), Ribo-Zero™ rRNA Removal Kit (Plant Leaf) and Ribo-Zero™ rRNA Removal Kit (Bacteria) (Illumina, San Diego, CA, USA). Using rRNA removal instead of oligo(dT)-based mRNA enrichment yielded mRNAs from both prokaryotes and eukaryotes. The purified mRNA was then fragmented with Elute, Prime, Fragment Mix. First-strand cDNA was synthesized using the First Strand Master Mix and Super Script II Reverse Transcriptase (Invitrogen, CA, USA). After purifying the product (Agencourt RNA Clean XP Beads, AGENCOURT; Beckman Coulter Genomics, Danvers, MA, USA), the second-strand cDNA was synthesized by adding Second Strand Master Mix and dATP, dGTP, dCTP, dUTP mix. The double-strand cDNA was processed by purification, end repair, and adaptor ligation. Fragments, which were about 400 bp (insert size about 250 bp), were selected and sequenced on the Illumina HiSeq 4000 instrument (Illumina, San Diego, CA, USA). Finally, Illumina high-throughput sequencing of all samples yielded a total of 881 Gb raw reads.

### Bioinformatic analysis and expression quantification of rhodopsins from the metatranscriptomes

After trimming adaptors from raw reads, sequences with >5% ambiguous bases (N) and low-quality reads (>20% bases with quality value < 20) were removed to obtain clean reads using Soapnuke (version 1.5.6). De novo assembly was carried out for remaining clean reads using Trinity, then Tgicl was used to cluster transcripts to unigenes with a minimum of 95% identity between the contigs [44]. The unigene sets from all samples were merged to generate the final unigene dataset (Unigene) for downstream analysis. The taxonomic were analyzed using BLASTX base on NR and BLASTN base on Nucleotide Squence Database (NT) (version 20180814) with the following cutoff values: E-value < 10^−5^ and identity >40%. The best hit with strong e value was assigned the organism from which the microbial rhodopsins sequence was originated. SwissProt functional annotation was conducted using Diamond BLASTX [45]. Bowtie2 [46] was used to align clean reads to the unigene dataset (as reference), and then Salmon v0.9.1 [47] was used to calculate gene expression levels in each sample. In the subsequent analysis, we eliminated unigenes whose TPM (Transcripts Per Kilobase of exon model per Million mapped reads) was less than 0.1 across all 20 samples.

### Phylogenetic analysis

The phylogenetic analysis was conducted to assess the diversity and classification of rhodopsins (PPRs and other types of rhodopsins). We chose the sequences from our metatranscriptome data that at least contained transmembrane helices C-F. Deduced protein sequences and selected reference sequences from the NCBI database were aligned using the Muscle model in MEGA X [48] (Supplementary file 3). Model Test in MEGA X was used to find the best model of amino acid evolution. The phylogenetic tree was inferred using Maximum likelihood method in MEGA X using LG + G model with 1000 bootstrap replicates performed to obtain statistical support for the tree topology. After exporting the tree in the Newick format, color modification of phylogenetic trees was added using iTOL.

### Statistical analysis

#### Merger of datasets from size fractions to enable comparison

Large (3–200 µm) and small (0.2–3 µm) size fractions samples were from the same water samples even though they were sequenced separately. To facilitate the comparison of prokaryotic and eukaryotic contributions to the rhodopsin mRNA pool in each water sample, the data from the two size fractions were combined after adjusted for the volume of the water sample. We multiplied the TPMs of prokaryotic (Pro-TPM) or eukaryotic (Euk-TPM) rhodopsins in the small and large size fractions with their respective amounts of RNA extracted from the size fraction samples, respectively, added the two products, and then divided the sum by the total RNA mass extracted from both size fractions from the same volume of water sample, i.e. Rhodopsin contributions of prokaryotic microbes = (Pro-TPMsmall * RNAsmall per L+ Pro-TPMlarge * RNAlarge per L)/(RNAsmall per L + RNAlarge per L) and the Rhodopsin contributions of eukaryotic microbes = (Euk-TPMsmall * RNAsmall per L + Euk-TPMlarge * RNAlarge per L)/(RNAsmall per L + RNAlarge per L). These allow estimation of contribution of Prokaryotic (Pro) or Eukaryotic (Euk) microbes from both size fractions to the total rhodopsin transcript pool from each plankton community (water sample).

#### Distance correlations between microbial rhodopsin and environmental factors

We computed pairwise distances between samples based on microbial rhodopsin or PPRs abundances (TPM) matrix, from prokaryotic or eukaryotic microbes, and environmental factors matrix. The following environmental factors were chosen for correlation analysis: NO_2_^-^ (µmol/L), PO_4_^3-^ (µmol/L), NO_3_^-^_NO_2_^-^ (nitrate plus nitrite, µmol/L), SiO_3_^2-^ (µmol/L), depth, temperature, salinity, and N:P ratio. These distance matrices were computed using partial Mantel correlations in R studio through the vegan R software package.

#### Difference and correlation analysis

To evaluate the statistical significance of the differences observed between different sampling sites, size fractions or water depths, the analysis of variance (ANOVA) was performed using the IBM SPSS statistics package (version: 18). All data presented in this study are means with standard deviation calculated from the duplicate samples in each condition. The linear correlation between PPR expression and the abundance of source microbes was based on the Pearson correlation coefficient.

## Results

### Diversity of microbial rhodopsins

As mentioned earlier, microbial community structures based on SSU (rDNA) have been reported elsewhere [42, 43] and data are available (BioProject numbers PRJNA782430 and Accession number CNP0001483). The high-throughput sequencing of our RNA samples yielded 881 Gb raw data, which resulted in 792.7 Gb of clean reads in total after quality processing. These reads were assembled into 4,499,414 unigenes with N50 of 372 bp and maximum length 71,092 bp. After assembling, the computational pooling and clustering of all rhodopsin cDNA sequences from our metatranscriptomes yielded 1,765 rhodopsin unigenes.

**Fig. 1:**
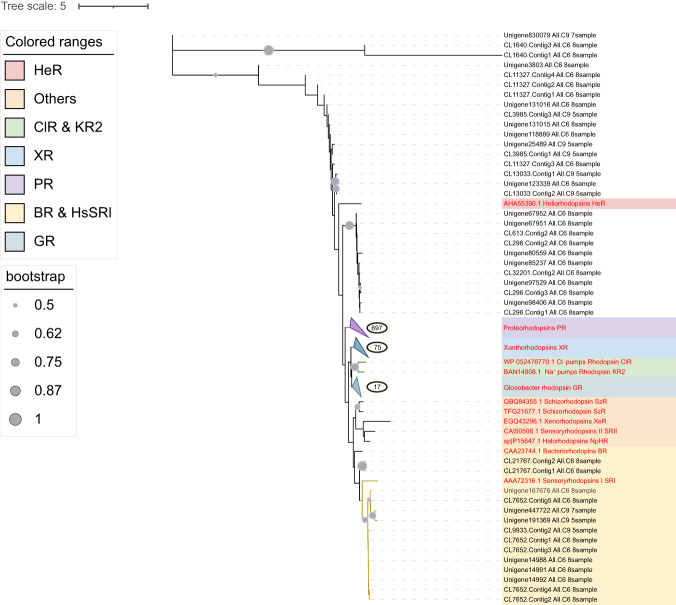
Maximum likelihood phylogenetic tree of microbial rhodopsin amino acid sequences. Different colors show types of rhodopsin, which are represented by reference sequences marked in red. Types of rhodopsin are shown on the medial left. The tree scale is shown on the upper left. Bootstrap is shown on the lower left. The number in the circles depicts the total number of rhodopsin unigenes in the cluster. Genbank accession number of reference PRs, XRs and GRs are as follows: PRs (AAG10475.1, AAK30179.1, BAN14807.1, AAZ21446.1), XRs (AIN36550.1, ADY17811.1, ADY17809.1, ADY17808.1, ABV22426.1, ABV22432.1, AAO14677.1, AEF32711.1, ABV22427.1, ADY17806.1, AJA37445.1, WP_011404249.1, AEP68177.1, AKG94905.1), GR (BAC88139.1).

To classify these sequences into the existing rhodopsin classification scheme, we constructed a phylogenetic tree using deduced amino acid sequences long enough to include helices C-F (totally 831 unigenes), with reference sequences from NCBI to represent existing rhodopsin groups. Phylogenetic analysis results showed that the cluster of proteorhodopsins (PRs) contained the highest number of rhodopsin unigenes, followed by xanthorhodopsins (XR and GR in the phylogenetic tree) and bacteriorhodopsin-like proteins (including BR and SRI in the phylogenetic tree) (Fig. 1). Nearly all of these are light-driven outward proton pump rhodopsins (i.e. ATP producing type) or PPRs for brevity. In the phylogenetic tree, we also found several unigenes that were affiliated with sensory rhodopsin-I, which is non-proton pump microbial rhodopsin. Besides, according to the SwissProt annotation results, other non-proton pump rhodopsin existed in the metatranscriptomes, including Archaerhodopsin from *Halobacterium* sp. and *Halorubrum chaoviator*, Cruxrhodopsin from *Haloarcula argentinensis*, sensory rhodopsin-II from *Haloarcula vallismortis*, and Octopus rhodopsin from *Enteroctopus dofleini*. However, these many classification types of rhodopsin exhibited low expression levels (Table 1).

**Table 1:** Expression levels ranges of different rhodopsins based on SwissProt annotation results.

Rhodopsin types	C6 (TPM ± SD^a^)	C9 (TPM ± SD)
Proteorhodopsin & Xanthorhodopsin	931–6413 (±20–±2230)	5–4282 (±2–±2215)
Bacteriorhodopsin	2–14 (±0–±2)	0–2 (±0–±4)
Sensoryrhodopsin	0.43–1 (±0–±1)	0–1 (±0–±2)
Archaerhodopsin	3–24 (±0–±7)	0–9 (±0–±12)
Cruxrhodopsin	0–3 (±0–±4)	0–0.3 (±0–±0.4)
rhodopsin from *Enteroctopus dofleini*	0	0–2 (±0–±2)

SD^a^: Corresponding ranges of standard deviation.

### Spatial and taxonomic differences in transcript abundance of microbial rhodopsin

**Fig. 2:**
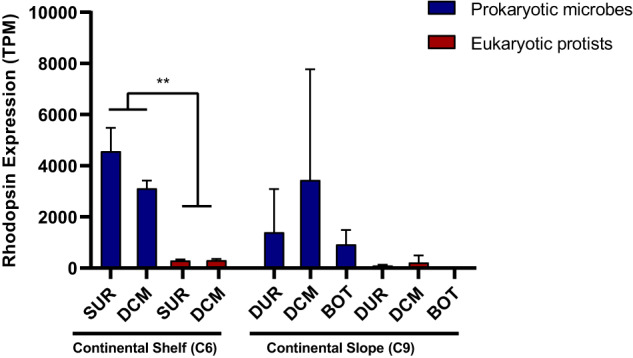
Rhodopsin expression levels in prokaryotic and eukaryotic microbes at different depths. SUR surface water, DCM deep chlorophyll maximum, BOT bottom of photic zone.

**Fig. 3:**
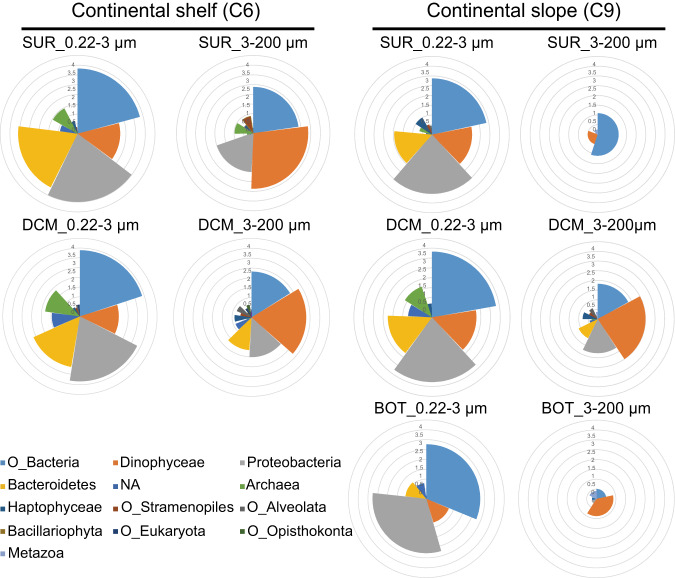
Expression levels of rhodopsin in different size fractions at different depths at shelf and slope sites. In a Nightingale rose diagram, different colors indicate rhodopsins from different supergroups of microbes. Radius of sectors show expression level in each supergroup of microbe. Central angle shows proportion of each microbial supergroup’s rhodopsin transcript in all microbial groups’ rhodopsin transcripts. SUR surface water, DCM deep chlorophyll maximum, BOT bottom of photic zone.

The taxonomic origin of the microbial rhodopsins detected in the samples was determined from metatranscriptome annotation against NCBI databases. The result provided a clear separation of prokaryotic and eukaryotic rhodopsins and assigned the sequences to specific taxa. In all our metatranscriptomic datasets combined, prokaryotic microbes accounted for 71% of the rhodopsin unigenes number and 40–97% of the rhodopsin contribution whereas microeukaryotes (protists) accounted for 27% of unigenes number and 2–60% of contribution, respectively. We then counted all the microbial rhodopsins expressed by prokaryotic and eukaryotic microbes in both the small (0.2–3 μm) and large size fraction (3–200 μm) and compared station- and lineage-wise differences using non-parametric ANOVA. In the transcriptomic data of shelf station (C6) samples, rhodopsin contribution in prokaryotic microbes were more abundant than those in protists (*p* < 0.05, Fig. 2). However, in slope station (C9) samples, there was no significant difference between prokaryotes and eukaryotes in the rhodopsin contribution due to large variations between samples (*p* > 0.05, Fig. 2). In addition, between the two study sites, the contribution of total microbial rhodopsins at the continental shelf station appeared to be slightly higher than the continental slope station but without statistical significance, regardless of size fractions (Figs. 2 and 3).

To examine different microbe supergroups’ contribution to rhodopsin expression, the expression level (TPM) of rhodopsin transcripts in different size fractions at different samples were analyzed. According to taxonomic annotation against NCBI databases, in the small-sized samples, rhodopsins were mainly expressed by Proteobacteria, Bacteroidetes, and Other_Bacteria, whereas in large-sized samples, rhodopsins were dominantly expressed by Dinophyceae, Proteobacteria, and Other_Bacteria (Fig. 3). The proteobacteria and Other_Bacteria in the large-sized fraction should be associated, endosymbiotically or ectosymbiotically, with eukaryotes, although some of them might be free-living bacteria retained on filters because of clogging during filtration.

**Fig. 4:**
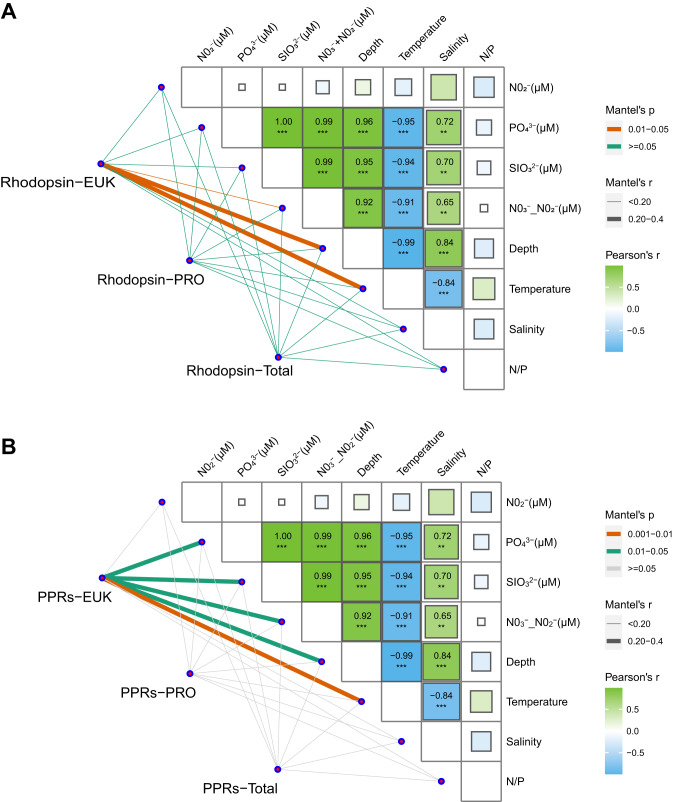
Correlations between microbial rhodopsin or PPRs and environmental factors. **A** The correlations between microbial rhodopsin and environmental factors. The Rhodopsin-EUK, Rhodopsin-PRO and Rhodopsin-Total were the abundances (TPM) of all rhodopsin annotated to eukaryotic, prokaryotic microbes or total microbial rhodopsins mRNA pool, respectively. **B** The correlations between PPRs and environmental factors. PPRs-EUK and PPRs-PRO were the abundances of PPRs, including PRs, BRs, and XRs from prokaryotic and eukaryotic microbes, respectively. PPRs-Total is the abundance (TPM) of all PPRs from prokaryotic and eukaryotic microbes. Edge width corresponds to the Mantel’s r statistic for the corresponding distance correlations. The sizes of boxes and the number in the boxes signify the Pearson correlation coefficient between environmental parameters.

According to the results of the Mantel test, the transcript abundance of total microbial rhodopsin from protists was correlated with depth and temperature (Fig. 4A), whereas the transcript abundance of protist PPRs was correlated with nutrient concentrations, including phosphate (PO_4_^3-^), nitrate plus nitrite (NO_3_^-^_NO_2_^-^), and silicate (SiO_3_^2-^), besides depth and temperature (Fig. 4B). In contrast, transcript abundances of prokaryotic rhodopsin and total rhodopsin (prokaryotic and eukaryotic combined) showed weak correlation with environmental parameters.

### The distribution of blue light- and green light-absorbing PPRs

**Fig. 5:**
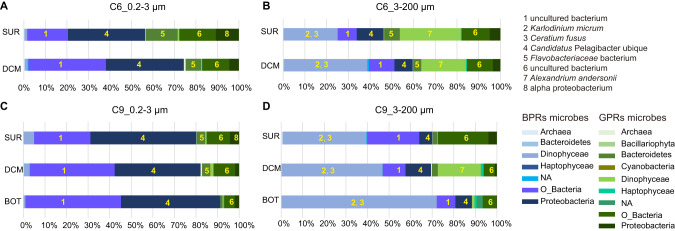
The distribution of expressed BPRs and GPRs in different size fractions at different depths at two study sites. **A**, **B** Shelf station; **C**, **D** slope station. The different shades of blue and green boxes depict different BPRs and GPRs expressed by different supergroups of microbes. Numbers in boxes indicate the highest (or top two) expressing species (shown on the upper right) in its supergroup (shown on the lower right). Labels on the left of each row show sampling depth: SUR surface water, DCM deep chlorophyll maximum, BOT bottom of photic zone.

Blue light-absorbing PPRs (BPRs) contain the polar glutamine at the spectrum tuning site (105 of the typical PPR), equivalent to position 104 in the PPR of *P. shikokuense*, whereas green light-absorbing PPRs (GPRs) contain methionine or leucine at that position [24, 39, 40]. We identified and counted BPR and GPR of all unigenes in the microbial rhodopsin pool based on the functional annotation results against SwissProt datasets. For both shelf and slope stations, the expression of BPRs increased with the increase of depth, whereas the expression of GPRs decreased with increasing depth (Fig. 5). GPRs were mainly distributed in large-sized microorganisms of the surface assemblages at the shelf station. In contrast, BPRs were dominant in small-sized microorganisms of both study sites especially deeper depths at the slope station (Fig. 5).

**Fig. 6:**
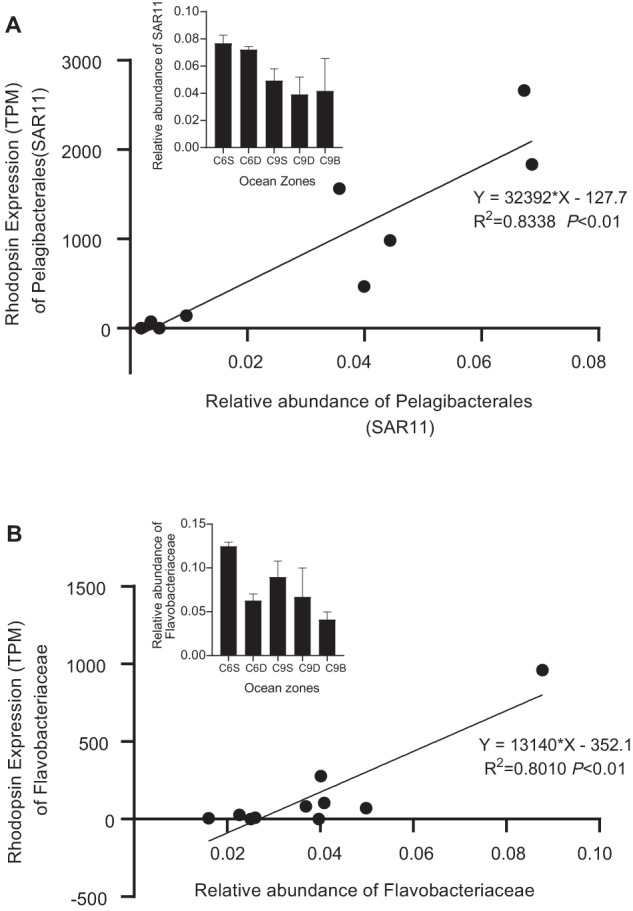
Correlation between PPR expression in the abundance of source microbes. **A** Pelagibacterales, which is represented by SAR11; **B** Flavobacteriaceae. Insets show the abundance of the organisms in different samples.

Based on annotation results of function and taxonomy, the PPRs of prokaryotes we detected were predominantly blue light-absorbing and were primarily contributed by *Candidatus* Pelagibacter ubique and some uncultured bacteria (Fig. 5). *Cand*. P. ubique is a member of the Pelagibacterales (SAR11). The BPR transcript abundance of Pelagibacterales was strongly correlated with the relative abundance of Pelagibacterales (SAR11) based on 16 S rRNA gene data (Fig. 6A, R^2^ = 0.8338). Meanwhile, GPRs also occurred in prokaryotes, primarily contributed by Flavobacteriaceae (Fig. 5). Similar to the case of BPR in Pelagibacterales, the GPR transcript abundance in Flavobacteriaceae also exhibited a strong correlation with the relative abundance of Flavobacteriaceae based on 16 S rRNA gene data (Fig. 6B, R^2^ = 0.801). For protists, our metatranscriptome data showed that their PPRs were dominantly blue light-absorbing and were mainly harbored and expressed by the dinoflagellate species annotated as *Karlodinium micrum*, and *Ceratium fusus*, whereas their green light-absorbing rhodopsin mainly expressed by *Alexandrium andersonii* (Fig. 5), indicating that dinoflagellates were the major contributors of eukaryotic protists’ PPRs in the study area.

## Discussion

### The dominance of ATP-producing PPRs in the microbial rhodopsin landscape

To explore relationship between microbes and corresponding rhodopsin expression, we portrayed a microbial rhodopsin landscape using the RNA-seq method instead of rhodopsin PCR amplification to avoid potential PCR biases. In the small-sized samples, rhodopsins expression level was the highest in Proteobacteria whereas in large-sized samples rhodopsins were dominantly expressed by Dinophyceae (Fig. 3). At the same time, according to 16 S and 18 S rDNA sequencing results, at the phylum/class level, Dinophyceae was the most abundantly represented among eukaryotic microbes in our samples (Fig. S2A), while proteobacteria were the most abundant prokaryotic phylum in microbial communities of the two study sites (Fig. S2B). However, we must note that, as is true in any rDNA-based metabarcoding studies, the abundance of the barcode (marker gene) does not directly represent the abundance of the cells, because rDNA copy number per cell varies between lineages, and is particularly high in dinoflagellates [49]. Therefore, the corresponding trends of PPR expression and rDNA abundance for Proteobacteria and Dinophyceae suggest the possibility that PPR promotes the growth of these lineages, but this still needs to be verified in the future using cell abundance data.

In total, seven types of rhodopsin (PR, BR, XR, Archaerhodopsin, Cruxrhodopsin, sensory rhodopsin, and Octopus rhodopsin) were detected in this study, but each contributed to the rhodopsin mRNA pool at very different levels. In the microbial rhodopsin pool, PPRs accounted for 16–98% of the total microbial rhodopsins mRNA pool in different samples. The PPRs in our study sites were mainly PRs and XRs, followed by BR-like proteins (Fig. 1 and Table 1), all of which are assumed to be light-driven outward proton pump rhodopsins that can presumably fuel ATP production. This is the first documentation of the rhodopsin profile in the NSCS and one of the few in the global ocean that compared the abundances of rhodopsin between prokaryotes and eukaryotes. More recently, two new types of PPRs, xenorhodopsins and schizorhodopsins, were discovered, which pump protons inward [19–21], but its spatial and taxonomic distribution is less clear. However, the inward proton pump rhodopsins were not found in the mRNA pool in the present study.

### Lineage- and space-differential distribution of expressed PPR

Between the two study sites, based on relative read counts, there were higher rhodopsin transcript abundances in the continental shelf region (C6) than the slope region (C9) (Fig. 1 and Table 1). Furthermore, in the transcriptomic data of shelf samples, the contribution of PPRs mRNA in prokaryotic microbes from both the small and large size fractions were higher than microeukaryotes (*p* < 0.05, Fig. 2), indicating that the prokaryotic microbes were more important contributors of rhodopsin-based solar energy converting mechanism than protists in the continental shelf region. However, in our transcripts data of slope samples, there was no significant difference in the abundance of rhodopsin mRNA between prokaryotic microbes and protists, indicating that in the continental slope region, the PPR-based energy harvesting mechanism was probably equally important for both prokaryotes and protists. Many of these protists are eukaryotic microalgae (phytoplankton) (Figs. 3 and 5), and thus possess not only a rhodopsin-based energy transducer system but also chlorophyll-*a*-based photosynthesis system, uniquely having two energy-harvesting mechanisms, or “dual engines.” The coexistence of rhodopsin and photosynthesis system might be important for protists inhabiting the more nutrient-poorer environment, which happened to be at the continental shelf station we sampled in the NSCS, as our nutrient data indicated (see below).

### Relationship between expressed PPR and nutrient condition

Previous studies have shown that the PPR-based energy mechanism may serve to ameliorate the deficiencies of nutrients such as nitrogen, phosphorus, or iron [18, 33, 36, 50]. For marine phytoplankton, nitrogen limitation depresses photosynthesis and growth as nitrogen is required for the synthesis of nucleic acid, protein, and Chl *a*; however, the precursor of all-*trans* retinal (functional equivalent of Chl *a*) is not affected by nutrient limitation [51, 52]. Therefore, PPRs might provide supplemental energy to enable the microbial community to survive in the nitrogen-limited condition.

At both study sites in the present study, nitrogen and phosphorus nutrient concentrations at surface layer were 0.156–0.607 μmol/L and 0.021–0.058 μmol/L respectively, with an N: P ratio of <12.7 (Supplementary file 2), lower than the typical Redfield ratio of 16:1, suggesting nitrogen limitation. Between the two stations, the average DIN concentration and N: P ratio at shelf region were lower than that at slope region (Supplementary file 2), and consistent with the expectation from the previous studies, a higher PPR mRNA abundance was found in microeukaryotes at shelf region. This PPR-nutrient relationship was further supported by the significant correlation found between the transcript abundance of microeukaryotic PPRs and nutrient concentrations and the lack of similar correlations for prokaryotic PPRs (Fig. 4A, B). The microeukaryotes are likely phytoplankton, which require nutrients for photosynthesis, unlike prokaryotes that require organic matter as source of nutrition.

For the eukaryotic microalgae (phytoplankton) that possess the “dual engines,” the supplemental energy provided by PPRs can probably support their carbon dioxide assimilation [38] and confer these organisms with competitive advantages in a nutrient stress environment. PPRs in diatoms are postulated to enable these diatoms to survive iron deficiency [18]. However, this function may not be as important in our study sites because surface water of the NSCS has been reported to be not iron limited [53].

In the PPR-carrying microorganisms, PPRs may enhance the fitness in the low dissolved organic carbon (DOC) environment [54]. In the present study, Flavobacteriaceae and *Candidatus* Pelagibacter ubique taxa were the major microbes that contained rhodopsin, GPR and BPR, respectively. Flavobacteria play a vital role in mineralizing DOC in the ocean. On the one hand, our results showed that PPR expression in Flavobacteriaceae was strongly correlated with the relative abundance of Flavobacteriaceae (Fig. 6B, R^2^ = 0.801), indicating the potential that PPR supported the growth of these bacteria. On the other hand, *Cand*. P. ubique is a species of the SAR11 clade, the most abundant group of heterotrophic bacteria in the ocean [55, 56], relying on PPR to support its endogenous carbon respiration when facing carbon starvation [57]. Its PPR expression was also strongly correlated with its relative abundance (Fig. 6A, R^2^ = 0.8338). In *Cand*. P. ubique, the relative PPR transcript abundance was higher at slope region than shelf region (Fig. 5), and consistent with the notion that PPR is selected for in nutrient-poor environments, DOC concentration has been reported to be lower at slope than shelf [58]. PPR might help increase the fitness of *Cand*. P. ubique in a low DOC environment.

### Differential absorbance spectrum shift and niche differentiation

The depth distribution of different spectrum absorbing rhodopsins has been shown to be related to light distribution characteristics [40]. In our results, GPRs were highly abundant both in the number of unigenes and in mRNA quantity in large-sized organisms in the surface water at both stations, especially at the shelf station, whereas BPRs were dominant in small-sized microorganisms in the deeper water, especially at the continental slope station (Fig. 5). This pattern is generally consistent with the pattern of spectrum-differential attenuation of light in the water column (blue light penetrates deeper than green light), indicating an adaptive evolution of the microbes.

This evolutionary adaptation may explain the differential distribution of two important groups of bacteria. In our samples, Flavobacteriaceae was more abundant in the surface, and their PPRs were the green-light absorbing type. In contrast, the change in relative abundance of *Cand*. P. ubique at different water depths was smaller than Flavobacteriaceae (Insets of Fig. 6), mirroring the pattern of their PPRs (Fig. 5), which was more stable between surface and DCM in *Cand*. P. ubique than in Flavobacteriaceae. This coincides with the fact that PPR of *Cand*. P. ubique were blue light absorbing type, and blue light can reach deeper in the water column.

The spectrum shift analysis was based on the annotation results against the SwissProt database. However, this method is not perfect. For instance, the PPRs in *K. micrum* and *C. fusus* were annotated as GPR in SwissProt annotation results. Yet, these *K. micrum* and *C. fusus* PPRs are actually BPRs rather than GPRs [59]. We corrected their annotation to BPRs and the final results indicate that there were abundant BPRs in the large-sized fraction and dinoflagellates were the major contributors of eukaryotic protists’ BPRs in the study area (Fig. 5). Furthermore, dinoflagellates possess BPRs and GPRs in addition to Chl *a*, which possibly possess the ability to utilize slightly different wavelength than non-rhodopsin-carrying phytoplankton in the NSCS.

## Supplementary Information

Supplementary 1

Supplementary 2

Supplementary 3

## Data Availability

All datasets used in this article have been deposited in NCBI database (http://www.ncbi.nlm.nih.gov/) under the BioProject number PRJNA729123, PRJNA782430 and CNGB Sequence Archive (CNSA) of China National GeneBank DataBase (CNGBdb) (https://db.cngb.org/cnsa/) under the accession number CNP0001483.

## References

[1] Pinhassi J, DeLong EF, Beja O, Gonzalez JM, Pedros-Alio C. Marine bacterial and archaeal ion-pumping rhodopsins: genetic diversity, physiology, and ecology. Microbiol Mol Biol Rev. 2016;80:929–54. 27630250 10.1128/MMBR.00003-16 PMC5116876

[2] Oesterhelt D, Stoeckenius W. Rhodopsin-like protein from the purple membrane of *Halobacterium halobium*. Nat New Biol. 1971;233:149–52. 4940442 10.1038/newbio233149a0

[3] Matsuno-Yagi A, Mukohata Y. Two possible roles of bacteriorhodopsin; a comparative study of strains of *Halobacterium halobium* differing in pigmentation. Biochem Biophys Res Commun. 1977;78:237–43. 20882 10.1016/0006-291x(77)91245-1

[4] Schobert B, Lanyi JK. Halorhodopsin is a light-driven chloride pump. J Biol Chem. 1982;257:10306–13. 7107607

[5] Béja O, Aravind L, Koonin EV, Suzuki MT, Hadd A, Nguyen LP, et al. Bacterial rhodopsin: evidence for a new type of phototrophy in the sea. Science. 2000;289:1902–6. 10988064 10.1126/science.289.5486.1902

[6] Rozenberg A, Inoue K, Kandori H, Béjà O. Microbial rhodopsins: the last two decades. Annu Rev Microbiol. 2021;75:427–47. 34343014 10.1146/annurev-micro-031721-020452

[7] Finkel OM, Béjà O, Belkin S. Global abundance of microbial rhodopsins. ISME J. 2013;7:448–51. 23051692 10.1038/ismej.2012.112 PMC3554412

[8] Brindefalk B, Ekman M, Ininbergs K, Dupont CL, Yooseph S, Pinhassi J, et al. Distribution and expression of microbial rhodopsins in the Baltic Sea and adjacent waters. Environ Microbiol. 2016;18:4442–55. 27306515 10.1111/1462-2920.13407

[9] Campbell BJ, Waidner LA, Cottrell MT, Kirchman DL. Abundant proteorhodopsin genes in the North Atlantic Ocean. Environ Microbiol. 2008;10:99–109. 18211270 10.1111/j.1462-2920.2007.01436.x

[10] Sabehi G, Loy A, Jung K-H, Partha R, Spudich JL, Isaacson T, et al. New insights into metabolic properties of marine bacteria encoding proteorhodopsins. PLoS Biol. 2005;3:e273. 16008504 10.1371/journal.pbio.0030273 PMC1175822

[11] Guerrero LD, Vikram S, Makhalanyane TP, Cowan DA. Evidence of microbial rhodopsins in Antarctic Dry Valley edaphic systems. Environ Microbiol. 2017;19:3755–67. 28752953 10.1111/1462-2920.13877

[12] Rusch DB, Halpern AL, Sutton G, Heidelberg KB, Williamson S, Yooseph S, et al. The Sorcerer II Global Ocean Sampling expedition: northwest Atlantic through eastern tropical Pacific. PLoS Biol. 2007;5:e77. 17355176 10.1371/journal.pbio.0050077 PMC1821060

[13] Vader A, Laughinghouse HD, Griffiths C, Jakobsen KS, Gabrielsen TM. Proton-pumping rhodopsins are abundantly expressed by microbial eukaryotes in a high-Arctic fjord. Environ Microbiol. 2018;20:890–902. 29266690 10.1111/1462-2920.14035

[14] Lopez JL, Golemba M, Hernandez E, Lozada M, Dionisi H, Jansson JK, et al. Microbial and viral-like rhodopsins present in coastal marine sediments from four polar and subpolar regions. FEMS Microbiol Ecol. 2017;93:fiw216. 10.1093/femsec/fiw216 27815287

[15] Yutin N, Koonin EV. Proteorhodopsin genes in giant viruses. Biol Direct. 2012;7:1–6. 23036091 10.1186/1745-6150-7-34 PMC3500653

[16] Kandori H. Ion-pumping microbial rhodopsins. Front Mol Biosci. 2015;2:52. 26442282 10.3389/fmolb.2015.00052 PMC4585134

[17] Lin S, Zhang H, Zhuang Y, Tran B, Gill J. Spliced leader–based metatranscriptomic analyses lead to recognition of hidden genomic features in dinoflagellates. Proc Natl Acad Sci USA. 2010;107:20033–8. 21041634 10.1073/pnas.1007246107 PMC2993343

[18] Marchetti A, Catlett D, Hopkinson BM, Ellis K, Cassar N. Marine diatom proteorhodopsins and their potential role in coping with low iron availability. ISME J. 2015;9:2745–8. 26023874 10.1038/ismej.2015.74 PMC4817633

[19] Inoue K, Ito S, Kato Y, Nomura Y, Shibata M, Uchihashi T, et al. A natural light-driven inward proton pump. Nat Commun. 2016;7:13415. 27853152 10.1038/ncomms13415 PMC5118547

[20] Shevchenko V, Mager T, Kovalev K, Polovinkin V, Alekseev A, Juettner J, et al. Inward H^+^ pump xenorhodopsin: Mechanism and alternative optogenetic approach. Sci Adv. 2017;3:e1603187. 28948217 10.1126/sciadv.1603187 PMC5609834

[21] Inoue K, Tsunoda SP, Singh M, Tomida S, Hososhima S, Konno M, et al. Schizorhodopsins: a family of rhodopsins from Asgard archaea that function as light-driven inward H^+^ pumps. Sci Adv. 2020;6:eaaz2441. 32300653 10.1126/sciadv.aaz2441 PMC7148096

[22] Béja O, Spudich EN, Spudich JL, Leclerc M, DeLong EF. Proteorhodopsin phototrophy in the ocean. Nature. 2001;411:786–9. 11459054 10.1038/35081051

[23] Martinez A, Bradley A, Waldbauer J, Summons R, DeLong E. Proteorhodopsin photosystem gene expression enables photophosphorylation in a heterologous host. Proc Natl Acad Sci USA. 2007;104:5590–5. 17372221 10.1073/pnas.0611470104 PMC1838496

[24] Gomez-Consarnau L, Gonzalez JM, Coll-Llado M, Gourdon P, Pascher T, Neutze R, et al. Light stimulates growth of proteorhodopsin-containing marine Flavobacteria. Nature. 2007;445:210–3. 17215843 10.1038/nature05381

[25] Oesterhelt D, Stoeckenius W. Functions of a new photoreceptor membrane. Proc Natl Acad Sci USA. 1973;70:2853–7. 4517939 10.1073/pnas.70.10.2853 PMC427124

[26] Kim SH, Cho JC, Jung KH. Characterization of a Xanthorhodopsin-homologue from the North Pole. Rapid Commun Photoscience. 2013;2:60–63.

[27] Inoue K. Diversity, Mechanism, and Optogenetic Application of Light-Driven Ion Pump Rhodopsins. In: Yawo H, Kandori H, Koizumi A, Kageyama R (eds). *Optogenetics: Light-Sensing Proteins and Their Applications in Neuroscience and Beyond*. Springer Singapore: Singapore, 2021. pp 89-126. 10.1007/978-981-15-8763-4_6 33398809

[28] Siletsky SA, Lukashev EP, Mamedov MD, Borisov VB, Balashov SP, Dolgikh DA, et al. His57 controls the efficiency of ESR, a light-driven proton pump from *Exiguobacterium sibiricum* at low and high pH. Biochim Biophys Acta Bioenerg. 2021;1862:148328. 33075275 10.1016/j.bbabio.2020.148328

[29] Nakamura S, Kikukawa T, Tamogami J, Kamiya M, Aizawa T, Hahn MW, et al. Photochemical characterization of actinorhodopsin and its functional existence in the natural host. Biochim Biophys Acta Bioenerg. 2016;1857:1900–8. 10.1016/j.bbabio.2016.09.006 27659506

[30] Raven JA. Functional evolution of photochemical energy transformations in oxygen-producing organisms. Funct Plant Biol. 2009;36:505–15. 32688665 10.1071/FP09087

[31] Gómez-Consarnau L, Akram N, Lindell K, Pedersen A, Neutze R. Proteorhodopsin Phototrophy Promotes Survival of Marine Bacteria during. PLoS Biol. 2010;8:e1000358. 20436956 10.1371/journal.pbio.1000358 PMC2860489

[32] Guo Z, Zhang H, Lin S. Light-promoted rhodopsin expression and starvation survival in the marine dinoflagellate *Oxyrrhis marina*. PLoS ONE. 2014;9:e114941. 25506945 10.1371/journal.pone.0114941 PMC4266641

[33] Shi X, Li L, Guo C, Lin X, Li M, Lin S. Rhodopsin gene expression regulated by the light dark cycle, light spectrum and light intensity in the dinoflagellate *Prorocentrum*. Front Microbiol. 2015;6:555. 26082770 10.3389/fmicb.2015.00555 PMC4451421

[34] Zhang Y, Lin X, Shi X, Lin L, Luo H, Li L, et al. Metatranscriptomic signatures associated with phytoplankton regime shift from diatom dominance to a dinoflagellate bloom. Front Microbiol. 2019;10:590. 30967855 10.3389/fmicb.2019.00590 PMC6439486

[35] Yu L, Zhang Y, Li M, Wang C, Lin X, Li L, et al. Comparative metatranscriptomic profiling and microRNA sequencing to reveal active metabolic pathways associated with a dinoflagellate bloom. Sci Total Environ. 2020;699:134323. 31522044 10.1016/j.scitotenv.2019.134323

[36] Gómez-Consarnau L, Raven JA, Levine NM, Cutter LS, Wang D, Seegers B, et al. Microbial rhodopsins are major contributors to the solar energy captured in the sea. Sci Adv. 2019;5:eaaw8855. 31457093 10.1126/sciadv.aaw8855 PMC6685716

[37] Fuhrman JA, Schwalbach MS, Stingl U. Proteorhodopsins: an array of physiological roles? Nat Rev Microbiol. 2008;6:488–94. 18475306 10.1038/nrmicro1893

[38] Larkum AWD, Ritchie RJ, Raven JA. Living off the Sun: chlorophylls, bacteriochlorophylls and rhodopsins. Photosynthetica. 2018;56:11–43.

[39] Man D, Wang W, Sabehi G, Aravind L, Post AF, Massana R, et al. Diversification and spectral tuning in marine proteorhodopsins. EMBO J. 2003;22:1725–31. 12682005 10.1093/emboj/cdg183 PMC154475

[40] Pushkarev A, Hevroni G, Roitman S, Shim JG, Choi A, Jung KH, et al. The use of a chimeric rhodopsin vector for the detection of new proteorhodopsins based on color. Front Microbiol. 2018;9:439. 29593685 10.3389/fmicb.2018.00439 PMC5859045

[41] Shim JG, Kang NR, Chuon K, Cho SG, Meas S, Jung KH. Mutational analyses identify a single amino acid critical for colour tuning in proteorhodopsins. FEBS Lett. 2022;596:784–95. 35090057 10.1002/1873-3468.14297

[42] Yuan H, Li T, Li H, Wang C, Li L, Lin X, et al. Diversity Distribution, Driving Factors and Assembly Mechanisms of Free-Living and Particle-Associated Bacterial Communities at a Subtropical Marginal Sea. Microorganisms. 2021;9:2445. 34946047 10.3390/microorganisms9122445 PMC8704526

[43] Lin S, Li T, Yuan H, Li H, Yu L, Zhuang Y, et al. Sediment trap study reveals dominant contribution of metazoans and dinoflagellates to carbon export and dynamic impacts of microbes in a subtropical marginal sea. J Geophys Res: Biogeosci. 2022;127:e2021JG006695.

[44] Pertea G, Huang X, Liang F, Antonescu V, Sultana R, Karamycheva S, et al. TIGR Gene Indices clustering tools (TGICL): a software system for fast clustering of large EST datasets. Bioinformatics. 2003;19:651–2. 12651724 10.1093/bioinformatics/btg034

[45] Buchfink B, Xie C, Huson DH. Fast and sensitive protein alignment using DIAMOND. Nat Methods. 2015;12:59–60. 25402007 10.1038/nmeth.3176

[46] Langmead B, Salzberg SL. Fast gapped-read alignment with Bowtie 2. Nat Methods. 2012;9:357–9. 22388286 10.1038/nmeth.1923 PMC3322381

[47] Patro R, Duggal G, Love MI, Irizarry RA, Kingsford C. Salmon provides fast and bias-aware quantification of transcript expression. Nat Methods. 2017;14:417–9. 28263959 10.1038/nmeth.4197 PMC5600148

[48] Kumar S, Stecher G, Li M, Knyaz C, Tamura K. MEGA X: molecular evolutionary genetics analysis across computing platforms. Mol Biol Evol. 2018;35:1547. 29722887 10.1093/molbev/msy096 PMC5967553

[49] Li T, Liu G, Yuan H, Chen J, Lin X, Li H, et al. Eukaryotic plankton community assembly and influencing factors between continental shelf and slope sites in the northern South China Sea. Environ Res. 2023;216:114584. 36270532 10.1016/j.envres.2022.114584

[50] Hassanzadeh B, Thomson B, Deans F, Wenley J, Lockwood S, Currie K, et al. Microbial rhodopsins are increasingly favoured over chlorophyll in High Nutrient Low Chlorophyll waters. Environ Microbiol Rep. 2021;13:401–6. 33870657 10.1111/1758-2229.12948

[51] Geider RJ, La Roche J, Greene RM, Olaizola M. Response of the photosynthetic apparatus of *Phaeodactylum tricornutum* (Bacillariophyceae) to nitrate, phosphate, or iron starvation^1^. J Phycol. 1993;29:755–66.

[52] Schlücker S, Szeghalmi A, Schmitt M, Popp J, Kiefer W. Density functional and vibrational spectroscopic analysis of *β*-carotene. J Raman Spectrosc. 2003;34:413–9.

[53] Zhang R, Zhu X, Yang C, Ye L, Zhang G, Ren J, et al. Distribution of dissolved iron in the Pearl River (Zhujiang) Estuary and the northern continental slope of the South China Sea. Deep Sea Res Part II. 2019;167:14–24.

[54] Palovaara J, Akram N, Baltar F, Bunse C, Forsberg J, Pedros-Alio C, et al. Stimulation of growth by proteorhodopsin phototrophy involves regulation of central metabolic pathways in marine planktonic bacteria. Proc Natl Acad Sci USA. 2014;111:E3650–3658. 25136122 10.1073/pnas.1402617111 PMC4156726

[55] Herlemann DP, Woelk J, Labrenz M, Jürgens K. Diversity and abundance of “Pelagibacterales”(SAR11) in the Baltic Sea salinity gradient. Syst Appl Microbiol. 2014;37:601–4. 25444644 10.1016/j.syapm.2014.09.002

[56] Durham BP, Boysen AK, Carlson LT, Groussman RD, Heal KR, Cain KR, et al. Sulfonate-based networks between eukaryotic phytoplankton and heterotrophic bacteria in the surface ocean. Nat Microbiol. 2019;4:1706–15. 31332382 10.1038/s41564-019-0507-5

[57] Steindler L, Schwalbach MS, Smith DP, Chan F, Giovannoni SJ. Energy starved *Candidatus* Pelagibacter ubique substitutes light-mediated ATP production for endogenous carbon respiration. PLoS ONE. 2011;6:e19725. 21573025 10.1371/journal.pone.0019725 PMC3090418

[58] Meng F, Dai M, Cao Z, Wu K, Zhao X, Li X, et al. Seasonal dynamics of dissolved organic carbon under complex circulation schemes on a large continental shelf: The Northern South China Sea. J Geophys Res: Oceans. 2017;122:9415–28.

[59] Vader A, Laughinghouse HD IV, Griffiths C, Jakobsen KS, Gabrielsen TM. Proton‐pumping rhodopsins are abundantly expressed by microbial eukaryotes in a high‐Arctic fjord. Environ Microbiol. 2018;20:890–902. 29266690 10.1111/1462-2920.14035

