# Supplementary material for: A comparative study reveals the relative importance of prokaryotic and eukaryotic proton pump rhodopsins in a subtropical marginal sea
Source: ISME Commun. 2023 Aug 18;3:79. doi: 10.1038/s43705-023-00292-y (PMC10439184; doi:10.1038/s43705-023-00292-y)
Supplement: Supplementary file 1 — Supplementary 1 [file 43705_2023_292_MOESM1_ESM.docx]

**Supplementary material for**

A comparative study reveals the relative importance of prokaryotic and eukaryotic proton pump rhodopsins in a subtropical marginal sea

**Minglei Ma^1^,** **Hongfei Li^1, 2^, Cong Wang^1^, Tangcheng Li^1, 3^, Jierui Wang^1^, Huatao Yuan^1^, Liying Yu^1, 4^, Jingtian Wang^1^, Ling Li^1^, Senjie Lin^1, 5, 6*^**

1. State Key Laboratory of Marine Environmental Science, College of Ocean and Earth Science, Xiamen University, Xiamen 361102, China;
2. National Engineering Research Center for Marine Aquaculture, Zhejiang Ocean University, Zhoushan 316022, China;
3. Biology Department and Institute of Marine Sciences, College of Science, Shantou University, Shantou 515063, China;
4. Central Laboratory, the Second Affiliated Hospital of Fujian Medical University, Quanzhou 362000, China
5. Laboratory of Marine Biology and Biotechnology, Qingdao National Laboratory of Marine Science and Technology, Qingdao 266237, China
6. Department of Marine Sciences, University of Connecticut, Groton CT 06340, USA;

***** **Correspondence:**

Corresponding Author

senjie.lin@uconn.edu

**Supplementary material 1**

**Fig. S1 Study sites in the northern South China Sea.** C6 in the continental shelf and C9 in the continental slope.

**
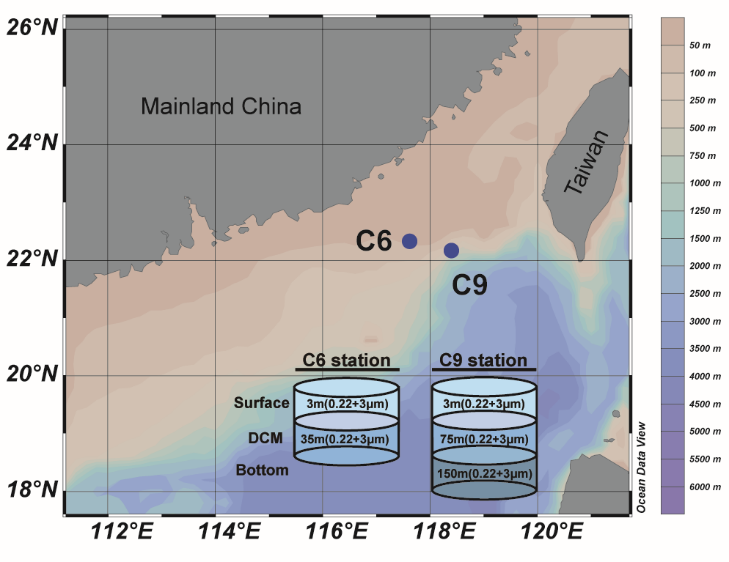
**

**Fig. S2 The main phyla/class microbes in C6 and C9.** (A) main eukaryotic microbes; (B) main prokaryotic microbes.**
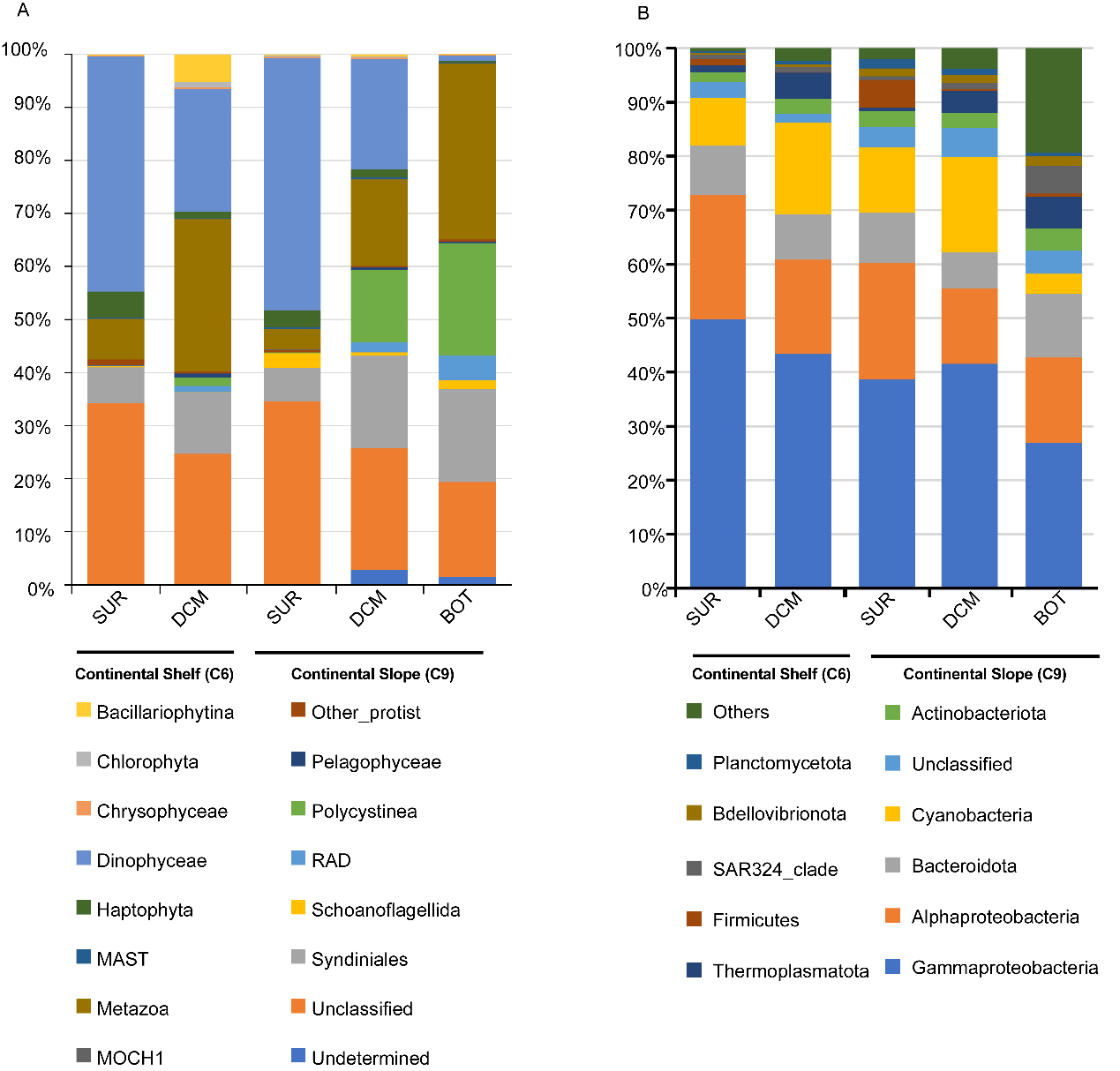
**
